# An Update on the Management of Bone Metastases

**DOI:** 10.1007/s11912-024-01515-8

**Published:** 2024-03-28

**Authors:** Alexander J. Grosinger, Sara R. Alcorn

**Affiliations:** Department of Radiation Oncology, University of Minnesota Medical School, Mail Code 494, 420 Delaware St. SE, Minneapolis, MN 55455-0110 USA

**Keywords:** Palliation, SBRT, Radiation, Bone metastases

## Abstract

**Purpose of Review:**

Increasing life expectancy among patients with advanced cancer has placed a greater emphasis on optimizing pain control and quality of life. Concurrently, significant advancements in radiotherapy for bone metastases have permitted for dose escalation strategies such as stereotactic radiotherapy. This review aims to provide updated information on the management of bone metastases in light of these developments.

**Recent Findings:**

We reviewed recent studies regarding the role and details of external beam radiotherapy for bone metastases, with emphasis on differences by treatment site as well as intention (palliative versus ablative for oligometastases). Conventional palliative radiotherapy remains a mainstay of management. While stereotactic radiotherapy may augment durability of pain relief and even survival time, there are significant questions remaining regarding optimal dosing and patient selection.

**Summary:**

Radiotherapy for bone metastases continues to evolve, particularly with increasing use of stereotactic radiotherapy. Future studies are needed to clarify optimal dose, fractionation, modality, and patient selection criteria among different radiotherapy approaches.

## Introduction

Metastases to bone are common in advanced cancer and are associated with significant morbidity through tumor-related pain, pathologic fractures, and neuraxis compromise. Palliative radiotherapy has been shown to be an effective management strategy for symptomatic bone metastases, affording a high degree of control of pain and other symptoms for patients [1•]. Given increasing life expectancy among patients with advanced cancer, there is a growing emphasis on controlling symptomatic bone metastases in order to preserve quality of life and perhaps to improve survival in select cases [2••]. Thus, while conventional palliative regimens are most commonly employed, advanced treatment approaches such as stereotactic body radiotherapy (SBRT) are increasingly utilized in an effort to augment durable disease outcomes, including in patients with oligometastatic disease [1•]. In this update, we will discuss recent studies describing palliative radiotherapy approaches for *symptomatic* bone metastases, focusing on treatment site and radiotherapy modality. Then we will review literature on radiotherapy for *oligometastatic* bone disease.

## Conventional Radiotherapy for Symptomatic Bone Metastases

### General Considerations for Single and Multiple-Fraction Conventional Radiotherapy

Numerous randomized studies have compared single- to multiple-fraction radiotherapy for the management of symptomatic bone metastases, with single-fraction regimens most commonly comprised of 8 Gy/1 fraction and multiple-fraction arms consisting of regimens such as 20 Gy/5 fractions, 24 Gy/6 fractions, and 30 Gy/10 fractions [3, 4]. In these studies, radiotherapy was generally delivered using 2D and 3D-conformal approaches, and most trials included only patients with “uncomplicated” bone metastases that lacked higher risk features such as impending or existing pathologic fracture as well as spinal cord or cauda equina compression [5].

Rich et al.’s updated systematic review of 29 such trials demonstrated equivalent levels of pain relief between single- and multiple-fraction radiotherapy, with overall response rates of 61% versus 62% and complete response rates of 23% versus 24%, respectively. In sensitivity analyses that excluded patients without follow-up information, Rich et al., noted a statistically significant difference in overall response rate which favored multiple-fraction radiotherapy, although the magnitude of the difference was small (74% versus 76%) [3]. While there was no significant difference between arms in terms of risk of pathologic fracture, spinal cord compression, or acute toxicities, retreatment rates were higher in the single-fraction arms (20% versus 8%) [3]. It is unclear if retreatment rates reflect differences in durability between regimens or greater comfortability with retreatment after single-fraction radiotherapy.

Important caveats limit uniform application of single-fraction regimens. Given that most trials included only patients with “uncomplicated” bone metastases, the impact of “complicating” features on clinical outcomes between regimens is unknown. One single-institution study reported that up to 2/3 of targeted symptomatic bone metastases may have “complicating” features such as a history of prior radiation or surgery, impending or existing pathologic fracture, soft tissue extraosseous component, or neuraxis compromise. As such, a majority of patients seen in clinical practice may have been excluded from data supporting single-fraction regimens. Among the trials studied in Rich et al.’s systematic review, a majority of patients had breast and prostate cancer [3], limiting application of this data for less common—and often more radioresistant—tumor types such as renal cell carcinomas, sarcomas, and melanomas. Moreover, patients with prolonged life expectancies may benefit from potential improvements to disease outcomes and durability associated with dose-escalation with stereotactic radiotherapy, as discussed in the sections below.

Consideration of the interplay of retreatment and cost-effectiveness may also impact fractionation decisions. Shorter regimens are generally considered to be more cost-effective and convenient for patients; among the above-noted randomized trials, economic analyses tend to favor single-fraction arms for uncomplicated metastases, even after consideration of retreatment costs [6–8]. Nongkynrih et al. assessed the socioeconomic impact of single- versus multiple-fraction palliative radiation for painful bone metastases at their regional cancer center in India, including consideration of factors such as travel distance to the treatment center [9]. In their setting, authors found that the cost-effectiveness of single-fraction treatment was overcome by the costs associated with higher retreatment rates. While 30 Gy/10 fractions and 20 Gy/5 fractions offered similar levels of palliation, travel costs were significantly less onerous with the shorter regimen. As such, Nongkynrih and colleagues concluded that 20 Gy/5 fractions may comprise the most cost effective regimen for their patients by balancing cost considerations. [9].

### Conventional Fractionation Schedules for Symptomatic Compressive Spinal Metastases

Malignant epidural spinal cord compression secondary to a spine metastasis are considered an indication for urgent invention. Radiation oncologists generally defer to open surgery to decompress the spine in patients who are surgical candidates, as this affords the greatest chance of regaining neurological function. In a landmark phase III trial, Patchell et al. compared cord decompression surgery and postoperative conventional radiotherapy (30 Gy/10 fractions) to radiotherapy alone (30 Gy/10 fractions) in patients with solid malignancies causing spinal cord compression. Inclusion criteria included (1) an estimated survival of > 3 months, (2) single level cord compression, with compression of the cauda equina excluded, (3) total paraplegia < 48 h, and (4) no prior radiation to the target area [10]. Authors found that combined open spine surgery with postoperative radiation conferred a statistically significant improvement in patients’ ability to retain or regain ambulatory function (84% versus 57%), maintain continence, and lower their daily pain medication and systemic steroid dose as compared to the radiotherapy only group [10]. This historic data strongly supports the role for surgical evaluation for all patients with symptomatic spinal cord compression who meet inclusion criteria for the trial.

For patients who are not optimal surgical candidates or who decline surgery, conventional radiotherapy alone is often considered for the management of symptomatic spinal cord or cauda equina compression. Treatment decisions in this clinical scenario are informed by the recent findings of the randomized SCORE-2 and SCORAD III trials [11–13]. In the SCORE-2 trial, Rades et al. demonstrated non-inferiority between 30 Gy/10 fractions and 20 Gy/5 fraction in patients with estimated life expectancy < 6 months, with 1-month overall response rates for motor function reported as 89.6% and 87.6%, respectively [11]. The SCORAD trial was a randomized trial that evaluated patients with a single site of cord compression who were treated with either 8 Gy/1 fraction or 20 Gy/5 fractions. At 8 weeks, the ability to walk (with or without ambulatory aids) was regained or retained by a similar proportion of patients in each arm (69.3% versus 72.7%, respectively). While the noninferiority margin between arms was not met, authors note that the absolute difference in ambulation rates were similar [13], supporting its use in patients with short survival time.

There are notable limitations to the application of data for conventional palliative radiotherapy in the setting of a spinal cord compression. For patients with neurological symptoms > 48 h or other exclusion criteria from the Patchell et al. study [10], the appropriateness of surgical intervention is ill-defined. Other considerations include tumor type, metastatic disease burden, and anticipated response to systemic therapy. In both the SCORE-2 and SCORAD trials, the overall survival time of included patients was approximately 3 months. This suggests that single- or 5-fraction treatment options may be best applied to patients with anticipated short life expectancy, as the higher biologically effective dose (BED) associated with 30 Gy/10 fractions may benefit patients with more prolonged life expectancy. Particular caution should be used when administering 8 Gy/1 fraction outside of a very limited life expectancy, given that the trial SCORAD did not confirm non-inferiority.

## SBRT for Symptomatic Bone Metastases

As previously noted, select patients with bone metastases may benefit from dose-escalation strategies. Dose escalation becomes important when accounting for the higher retreatment rates seen in lower dose, single-fraction conventional therapies and concerns over the durability of therapeutic effect in the context of protracted patient survival times, particularly at critical sites like the spine [2••]. Through the use of highly conformal techniques such as SBRT, dose-escalation strategies are being increasingly utilized. First, we will consider recent studies that compare SBRT versus conventional palliative radiotherapy in *symptomatic* non-spine or combined spine and non-spine metastases. In later sections, we will review the literature specific for SBRT in *symptomatic* spine metastases as well as in *asymptomatic/oligometastatic* settings.

### Palliative SBRT for Non-spine or Combined Spine and Non-spine Sites

While literature for SBRT in the setting of spine metastases is more robust, a growing body of evidence for SBRT to non-spine sites exists [2••]. We will review 2 randomized trials of SBRT versus conventional radiotherapy for the palliation of painful bone metastases of either non-spine or combination spine plus non-spine sites.

Nguyen and coauthors’ randomized phase II trial compared the analgesic durability of high dose SBRT (12 to 16 Gy, depending on the size of the lesion) to conventional multiple faction palliative radiotherapy (30 Gy/10 fractions) in patients with painful non-spine bone metastases. In the intent-to-treat analysis, they found SBRT to be noninferior to conventionally fractionated palliative doses for overall pain response up to 12 months, with pain response (complete or partial) rates of 44% versus 30% at 1 month and 40% versus 21% at 3 months for SBRT versus conventional RT, respectively. Further, retreatment rates, acute toxicity, and fracture rates were similar between arms. The pain response rate was higher for patients treated with 16 Gy SBRT (62%) compared to 12 Gy SBRT (30%) and 30 Gy/10 fractions (21%). Thus, authors attributed the enhanced clinical response to the higher BED with SBRT. Although overall survival was not significantly different between arms in this study, the authors notably recommended use of SBRT specifically in patients with excellent performance status, prolonged life expectancy, and limited disease burden, along with validation in a phase III clinical trial [2••].

In the randomized phase II VERTICAL Trial, Pielkenrood et al. investigated pain response for painful bone metastases of both spine and non-spine sites with SBRT (18 Gy/1 fraction, 30 Gy/3 fractions, and 35 Gy/5 fractions) versus conventional palliative radiotherapy (8 Gy/1 fraction, 20 Gy/5 fractions, and 30 Gy/10 fractions). Approximately 50% of patients were treated to spine and 50% to non-spine sites. Authors found no difference in pain response level, change to quality of life, or pain scores between SBRT and conventional radiotherapy. The authors reported that over one-quarter of their patients declined SBRT and that approximately 20% of patients who choose SBRT could not complete their treatment. As such, the study was underpowered due to the unexpectedly high rate of patients declining SBRT. A potential reason for this was logistic, as conventional therapy permitted for a generally shorter waiting period between consultation and treatment initiation [14]. A secondary analysis of the VERTICAL trial revealed that both conventional radiotherapy and SBRT led to comparable improvements in patient quality of life [15]. Thus when selecting patients for SBRT, providers should consider a patient’s “waiting time” between consultation and treatment.

There are significant caveats to standardized use of SBRT in the setting of symptomatic bone metastases. Studies of SBRT versus conventional radiotherapy tend to exclude patients with “complicated” bone metastases such as those with fracture or lesions requiring surgery. Given the potential for higher fracture risk with SBRT, the appropriate management of patients with impending or existing fractures with this technique is unclear. In general, inclusion criteria for these studies were similar to those that compared single- versus multiple-fraction conventional palliative radiotherapy. Thus, there is no strong evidence to guide patient selection between this range of techniques. The above trial data is limited to phase II studies, and larger phase III studies are still pending. A subsequent study by the VERTICAL group is testing 8 Gy/1 fraction with conventional palliative radiation as compared to 18 Gy/1 fraction with SBRT combined with conventional 8 Gy/1 fraction to the larger bone compartment containing the lesion [16]. The ongoing phase III ROBOMET randomized control trial is comparing overall pain response and durability of treatment between 8 Gy/1 fraction via 3DCRT and 20 Gy/1 fraction via SBRT [17]. It is noted that pending results from additional studies, the ESTRO-ACROP guidelines for radiotherapy in uncomplicated bone metastases does not support the routine use of SBRT in this setting, although the statement permits for consideration of use in select patients [18].

### Palliative SBRT for Spine-Only Metastases

Spine lesions account for approximately 70% of all bone metastases, and palliative radiotherapy is often employed to reduce pain, treat or prevent neuraxis compromise, and improve quality of life [19••]. To date, the optimal SBRT regimen is still under investigation, but common SBRT dosing regiments for spinal metastases include 16 − 24 Gy/1 Fraction, 24 Gy/ 2 fractions, 24 − 30 Gy/3 fractions, and 30 − 40 Gy/5 fractions. A recent literature review emphasizes that patient selection is the one of the most important factors for utilizing spine SBRT [20].

#### SBRT for Spinal Metastases Without Spinal Cord Compression

Sprave et al. conducted a randomized phase II study investigating 24 Gy/1 fraction via SBRT compared to 30 Gy/10 fractions via 3DCRT for spine metastases without spinal cord compression. While there was no difference in the primary outcome of pain relief > 2 points at 3 months, a significant difference in favor of SBRT was measurable by 6 months [21]. A subsequent analysis found a similar increase in bone density in the SBRT group compared to the 3DCRT group at 3 and 6 months, though there was a trend towards higher baseline pathologic fractures in the SBRT group [21, 22]. Given that fracture risk associated with SBRT delivered as 24 Gy/1 was reported as 28%, compared to 5% in the conventional radiotherapy arm, caution should be exercised when selecting this dose in clinical practice.

In the randomized SC24 Trial of spinal metastases without cord compression, Sahgal et al. demonstrated superior pain response with SBRT at 3 and 6 months in their comparison of SBRT (24Gy/2 fractions) and conventional radiotherapy (20 Gy/5 fractions) [19••]. Long-term follow-up demonstrated improved local control and lower rates of reirradiation in the SBRT group, with a shorter time to reirradiation in the conventional radiotherapy group [23]. Dunne et al. surmised that based on the SC24 trial, SBRT may be best suited for spinal metastases with extraosseous extensions, as approximately 60% of spinal targets were a “mass” type in each study arm [24].

In the most recent publication in this setting, Ryu et al. compared single doses of 16 or 18 Gy via SBRT to single doses of 8 Gy via conventional radiotherapy for spine metastases without neuraxis compression in a phase II/III randomized control trial, RTOG 0631. The authors did not find superiority of SBRT with regard to pain relief and suggested that pain responses were in fact worse within the first 3 months. The study also showed no significant control of typically radioresistant tumors compared to conventional radiotherapy [25•]. Sahgal and coauthors surmised that the differences in their outcomes compared to RTOG 0631 may be due to the higher BED used in the SC24 trial [19••]. Moreover, performance status was significantly lower in the SBRT arm, which was a covariate found to be associated with pain response. Given these findings, single-fraction SBRT doses of 16–18 Gy are not generally recommended.

While the SC24 regimen of 24 Gy/2 fractions is best supported by high-quality, randomized evidence for spinal metastases without spinal cord compression, additional SBRT fractionation regimens are described. Guckenberger et al. treated patients with spine metastases prospectively with 48.5 Gy/10 fractions or 35 Gy/5 fractions, reporting an overall pain response rate of 87% [26]. Long-term results showed a durable pain response at 5 years, in which 80% of reporting patients reported low levels of pain [27]. Zeng and coauthors demonstrated that dose escalating to 28 Gy/2 fractions was associated with improved local control rates without a sizable increase in risk of vertebral body compression fracture (10.7% at 2 years) [28].

Conflicting reports of the efficacy of SBRT for spine metastases without spinal cord compression noted above limit its clear application. This data suggests the need to define optimal dosing and fractionation with corresponding attention to dose constraints to minimize toxicity risk. Moreover, the studies’ patient inclusion criteria overlaps with the criteria used for trials of single- versus multiple-fraction conventional radiotherapy and highlights the need for future research to help risk-stratify patients for optimal management. Additionally, patients on reported trials were treated to 1–2 contiguous spine levels, suggesting that this approach may be inappropriate for larger treatment targets. Multidisciplinary input should also be considered, including assessment of stability of the spine for potential stabilization as per the Spinal Instability Neoplastic Scores (SINS) criteria when applicable [29].

#### SBRT for Spinal Metastases Causing Spinal Cord Compression

While surgical intervention is considered the standard of care for operative candidates meeting criteria for Patchell et al. [10], there is increasing interest in delivering SBRT to patients with spinal cord compression from bone metastases who do not meet these criteria or decline surgery.

At least 3 studies have investigated SBRT in the context of spinal cord compression for patients with minimal neurologic symptoms or who are not surgical candidates. Ryu et al. investigated SBRT treatments ranging from 12 to 20 Gy/1 fraction to treat patients with radiologic evidence of metastatic epidural compression but who had motor strength ≥ 4 out of 5 at presentation. Authors demonstrated an improvement in neurological function in 81% of cases, with a radiologic response rate of 80% for epidural disease at 2 months post-SBRT. No high-grade toxicity was reported [30]. In a phase III trial, ICORG 05-03, Lee et al. compared 10 Gy/1 fraction via SBRT with 20 Gy/5 fractions of conventional radiotherapy for patients with malignant spinal cord compression who were not candidates for surgical invention. They found a statistically significant improvement in quality of life with SBRT but no benefits for ambulation or pain control as compared with conventional radiotherapy [31]. In the phase II PRE-MODE trial, Rades et al. investigated 25 Gy/5 fractions with SBRT or IMRT for patients with spinal cord compression who were not surgical candidates and compared this to historic controls treated with conventional 20 Gy/5 fractions. The authors found that 25 Gy/5 fractions via SBRT or IMRT provided superior local progression free survival, motor deficit recovery and ambulatory recovery [32].

The use of spine SBRT in the postoperative setting is an area of additional ongoing investigation. Consensus guidelines by Redmond et al. suggest that postoperative spine SBRT may be most appropriate for patients with limited disease, radioresistant tumors, or in a salvage/re-irradiation setting [33]. Redmond et al. also investigated postoperative spine SBRT in which all patients received 35 Gy/5 fractions of SBRT within 16 weeks of spinal surgical resection. The authors found a > 90% local control rate at 1 year and that SBRT treatment resulted in downgraded epidural disease, particularly in patients who had residual high-grade epidural disease [34].

In addition to the above caveats for use of SBRT in spine metastases not causing spinal cord compression, limitations to standard use of spine SBRT in the setting of neuraxis compression includes the fairly preliminary nature of this data as well as the clear need for multidisciplinary input to determine surgical eligibility. Patients selected for SBRT in the above-noted trials were generally not considered to be surgical candidates, or in the case of Ryu et al., were minimally symptomatic from epidural spinal cord compression [30]. Moreover, it is noted that overall survival reported in the PRE-MODE trial was 3 months and was not reported in Ryu et al. or Lee et al. [31]. As such, applicability to patients with a more prolonged life expectancy is unclear.

### Systematic Reviews and Meta-analyses for SBRT versus Conventional Palliative Radiotherapy

Results and conclusions gleaned from systematic reviews and meta-analyses of SBRT versus conventional radiotherapy for symptomatic bone metastases are conflicting [35–37]. Four such meta-analyses consider the combination of spine and non-spine sites. Ito et al.’s meta-analysis demonstrated no statistical difference between conventional and SBRT for overall pain response, adverse events, quality of life or overall survival [35]. In contrast, Lee et al.’s meta-analysis demonstrated that SBRT reduced rates of progression, improved complete pain response rates at 3 months, and increases the likelihood of pain flares [36]. Song et al.’s meta-analysis concluded that SBRT had superior complete and partial response rates at 6 months and that SBRT is more optimally favored for oligometastatic disease, particularly for spine sites [37]. Song and coinvestgators also proposed that significantly increasing the BED of SBRT may have diminishing impact on the therapeutic effect of SBRT [37]. Lastly, Spencer and coauthors’ systemic review of palliative SBRT for bone metastases highlighted the importance and need for a consistent definition of pain response for palliative SBRT studies as well as the need to report pain response for all treated patients. Spencer and colleagues also stressed the importance of a consistent means of assessing radiographic response to palliative radiotherapy across all studies [38].

Specific to spine SBRT, a recent meta-analysis by Wong et al., concluded that there was no immediate benefit to quality of life, local progression or overall survival between SBRT versus conventional radiotherapy. However, Wong and coauthors postulated that SBRT could be associated with a significant improvement in pain over conventional radiotherapy at 3 and 6 months. The authors acknowledge the outsized contribution of RTOG 0631, which was a negative study with a large sample size [39]. However, as mentioned above, there are notable limitations to RTOG 0631 that may explain its null results.

## SBRT for Oligometastatic Disease

Oligometastatic disease, formally defined in 1995 as metastatic disease limited to a small number of foci due to anatomical and physiological factors, has been postulated to be amenable to curative radiotherapy, especially if treated with ablative doses prior to polymetastatic conversion [40–42]. The ESTRO and ASTRO consensus definition of oligometastatic disease is 1–5 metastatic foci, all of which can be safely treated with an optional controlled primary site [43].

In the phase II SABR-COMET trial of patients with well-controlled primary tumors of varying histology, < 5 oligometastatic foci were treated with SBRT schedules of 35 Gy/5 fractions, 60 Gy/8 fractions, and 54 Gy/3 fractions. Although bone metastases represented 35% of all metastatic sites treated, the SABR-COMET protocol specifically excluded femoral bone metastasis and metastatic foci within 3mm of the spinal cord [42]. At 5-year follow-up, researchers noted a median 22-month overall survival benefit for SBRT as compared to standard palliative treatment, with an absolute benefit of 25% at 5 years [44]. Currently, the phase III trials SABR-COMET-3 and SABR-COMET-10 are ongoing and assessing SBRT on overall survival in patients with a controlled primary solid tumor histology and up to 3 or 10 metastatic foci, respectively [45, 46]. Similar to SABR-COMET-3, the CORE Trial, a phase II/III trial, is assessing patients with a controlled primary tumor from breast, non-small cell lung cancer (NSCLC), or prostate cancer with up to three oligometastatic foci [44, 47].

Specifically for prostate cancer, both the STOMP and the ORIOLE trials have shown a benefit of SBRT to oligometastatic foci, as compared to observation of these lesions [48, 49]. The STOMP phase II trial demonstrated longer androgen deprivation therapy-free survival in patients with oligometastatic prostate cancer who were treated with 30 Gy/3 fractions via SBRT [49]. In the ORIOLE phase II trial, Phillips et al. demonstrated that treating three or fewer prostate oligometastatic bone foci with SBRT resulted in a one-third (19% versus 61%) reduction in both 6-month progression and 6-month radiographic progression on PSMA-PET (19% versus 63%) compared to observation, respectively [48].

For oligometastatic NSCLC, Gomez and colleagues conducted a phase II study evaluating patients with extracranial oligometastatic disease who were treated with both systemic and consolidative radiotherapy. More than half of patients received radiotherapy as SBRT or in a hypofractionated format. The study demonstrated that local consolidative radiotherapy extended progression free survival to approximately 12 months compared to approximately 4 months for patients only on maintenance treatment [50]. Long-term follow-up demonstrated an overall survival benefit as well [51]. Iyengar et al. completed a similar phase II study analyzing SBRT to treat oligometastatic NSCLC and also found a progression free survival benefit on the SBRT arm [52]. NRG LU-002 is an ongoing phase II trial assessing SBRT to treat oligometastatic NSCLC [47]. SARON is another, ongoing trial assessing post-chemotherapy SBRT to oligometastatic NSCLC with a primary aim of evaluating overall survival [47].

Specifically for breast cancer, Chmura et al. published their findings on treating oligometastatic breast cancer with SBRT on the phase II/III trial, NRG-BR002. Authors found that the addition of SBRT or metastatectomy to standard of care systemic therapy did not improve progression free survival or overall survival, and the trial did not proceed to a full phase III trial [53•].

Caveats to the standard use of SBRT for oligometastatic disease include the fairly preliminary nature of the data. Besides BR002, all of the aforementioned studies are phase II and would ideally be confirmed with a phase III randomized investigation. Moreover, the breadth of doses used on these studies suggests that optimal SBRT dosing and fractionation regimens as well as the optimal number of metastatic foci have yet to be determined. Given that many oligometastatic sites are asymptomatic, outcomes used elsewhere to assess the response to palliative radiotherapy may not be applicable. Due to differences in outcome and likely in patient survival, the doses used to treat oligometastatic disease may vary from those appropriate for the palliation of patients with more extensive or symptomatic metastatic disease burden. Present studies vary on the timing and extent of systemic therapy delivered prior to assessment for SBRT as well as with regard to details of primary site control.

## Ongoing and Future Research

### Prophylactic Radiotherapy for Bone Metastases

Traditionally, palliative radiotherapy has been withheld until a patient demonstrates bone metastasis-related symptoms. However, now that subsets of patients may be living long enough for skeletal related events (SRE, e.g., fracture, spinal cord compression or the need for surgery or radiotherapy to palliate symptoms) to develop, investigation of prophylactic radiotherapy to asymptomatic or minimally symptomatic bony lesions is needed [54, 55],

In a phase II clinical trial, Gillespie et al. investigated prophylactic radiotherapy to asymptomatic high-risk bone metastases, comparing results to systemic therapy or observation alone [56••]. High-risk lesions were defined as (1) bulky osseous lesions (≥ 2 cm); (2) disease involving the hip (acetabulum, femoral head, and femoral neck), shoulder (acromion, glenoid, and humeral head), or sacroiliac joints; (3) disease in long bones occupying one third to two third of the cortical thickness (humerus, radius, ulna, clavicle, femur, tibia, fibula, metacarpals, and phalanges); (4) disease in the vertebrae of the junctional spine (C7–T1, T12–L1, and L5–S1) and/or disease with posterior element involvement [55]. The most common prophylactic treatment regimens were 30 Gy/10 fractions, 27 Gy/3 fractions, 20 Gy/5 fractions, and 8 Gy/1 fraction, delivered via conventional radiotherapy or SBRT. At 1-year follow-up, there were significantly fewer SREs in the radiotherapy arm (1.6% versus 29%). There was also a significant association between SRE and poor overall survival, which the authors used to highlight and support prophylactic radiotherapy as a possible means of improving overall survival [56••].

There is an ongoing clinical trial investigating the utility of prioritizing prophylactic radiation in patients with bulky bony tumors or bony lesions in the junctional spine [56••]. Future efforts are required to better define the optimal dose and treatment approaches in this setting.

### Other Areas of On-going Research

A number of other areas of on-going and future research are notable in the management of bone metastases. Spatially fractionated radiation therapy, also known as GRID therapy, may provide a means to dose-escalate the treatment of bulky tumors, thus potentially improving local control while minimizing toxicity [57]. Combining radiotherapy with or sequential to ablative approaches such as cryoablation or heat-based ablation may also offer a promising means for escalating ablative management of bone metastases [58]. Given increased use of immunotherapy, research is required to define appropriate timing of palliative radiation relative to these and other systemic therapies.

## Conclusions

Recent technical advances in radiotherapy such as SBRT are changing the landscape of bone metastasis management and highlighting the role of dose-escalation and conformal strategies. While the use of advanced treatment techniques are promising, much of the data is fairly immature, and many questions regarding optimal dose, fractionation, modality, and patient selection criteria remain. Future studies should be aimed at addressing these concerns, with efforts made to standardize outcome measurements and assess patient preference and quality of life.

## Data Availability

No datasets were generated or analysed during the current study.
